# Progress in Surgery

**Published:** 1894-10-13

**Authors:** 


### Progress in Surgery.

### ABDOMINAL SURGERY.

Flateau[1] has abandoned the median incision in laparotomy. Out of thirty-three cases where the incision was extra-median, not one has been complicated by hernia of the cicatrix. He cuts two-fifths of an inch to the left of the linea alba, laying bare the rectus, the fibres of which are easily parted. He has never met with any difficulty in manipulating on the right side of the abdominal cavity, the incision being made on the left of the middle line. He uses braided silk for the sutures, which are entered close to the edge of the skin and include as much muscle and peritoneum as possible. In this way the turning in of the edges of the wound, which is apt to occur when a large tumour has been removed, is prevented. W. Fearnley[2] suggests that a rectangular flap of skin and subcutaneous tissue should be turned to the left, and the peritoneum then opened along the mid-line. As the flap itself is taken from the right side the peritoneum is well supported, after being stitched, by the undivided base of the flap.

Karl Able[3] reports a case of congenital defect of the diaphragm, with transference of the stomach, great omentum, part of the colon and duodenum into the left pleural cavity. The condition was diagnosed during life and operated on. The patient was a woman thirty-three years of age, who had borne seven children, and the hernia through the abnormal opening (which was seven centimetres long) in the left side of the central tendon, only commenced six days before death. The woman had taken a large quantity of beer and indigestible food. There was vomiting and pain in the abdomen, which commenced in the region of the stomach and extended to the back. The patient was lying in a flexed position on the left side, and vomiting was incessant. On the fourth day an attempt was made to pass a tube into the stomach, but an insuperable obstruction was met with in the cardiac region; the patient complained of great pain and became cyanotic. Some flatus was passed after an injection of linseed oil. On the fifth day the patient was collapsed, and the whole of the left side of the thorax tympanitic. The heart was displaced to the right, and the abdomen much retracted. The abdomen was opened on the sixth day by an incision running parallel with the left costal arch. The aperture in the diaphragm was found, and the greater part of the omentum, with the colon attached, was withdrawn from the thorax and replaced in the abdomen, but it was impossible to replace the stomach. The patient died three and a half hours afterwards. At the post-mortem examination the distended stomach occupied the whole of the left pleural sac. The left lung was compressed and airless. The œsophagus and duodenum were both tightly constricted. Dr. Able lays stress on the diagnostic importance of the retraction of the abdomen, the pain passing to the back, and the lateral position of the patient. The only reasonable treatment is operative.

H. Fischer,[4] in writing on suppurative inflammations in the sub-umbilical space, subdivides the peri-umbilical region into the supra-umbilical region, following the course of the vena umbilicalis or of the ligamentum suspensorium hepatis and the sub-umbilical region, which extends along the arteria umbilicalis and the space adjoining the urachus. These two areas are entirely separate, and yet inflammation in one may easily involve the other. His method of demonstration is by inserting the point of a syringe between the sheath of the rectus and the peritoneum, and slowly injecting coloured gelatin. As a result of this, a tumour shaped like the ace of spades, was produced, with its base at the navel and its apex six centimetres below the navel in the mid-line. The lateral portions were most protuberant, and the tumour decreased in prominence towards the mid-abdominal line, and along the linea alba was a deep groove. The measurements of the tumour were as follows: Breadth at base, 14·6 centimetres; breadth at apex, 1·6 centimetres; greatest length, 9 centimetres. Inflammation of this region may be acute or chronic, and may originate *in situ*, or occur from extension of the inflammatory process from a neighbouring locality.

1. *Acute Abscess of the Sub-umbilical Space.*—The author quotes five cases, all males, between seventeen and thirty-four years old, and with a previous healthy history. There was no history of injury in any case. A marked chill ushered in the attack in each case, and the temperature varied from 101 to 103 deg. Fahr. throughout the disease. The symptoms in the main were those of peritonitis, and continued to be alarming for two to four days, and then vomiting and collapse ceased, the bowels began to move, and the pain became localised about the umbilicus, with slight redness and marked œdema. Careful pulpation sometimes showed a firm area of infiltration, triangular in shape, and limited by the lateral recti boundaries, which muscles were moveable over the tumour. The infiltrated area could be lifted up with the abdominal wall. Fluctuation became more and more evident, and Fischer's cases were operated upon early in the second week. Yellow pus was evacuated, containing no gas and having no fæcal odour. The prognosis is favourable.

2. *Chronic Abscess in the Sub-umbilical Space.*—These are rarer than the acute variety, and in the majority of cases have been of tubercular origin. In a few cases actinomycosis abdominalis was the causative factor. There is no fever, and the course of the disease is a long one, and after the abscess has been emptied fistula may remain for a time, especially at the umbilicus.

3. *Rupture of Abscesses of Various Origin into the Sub-umbilical Space.*—An abscess of the liver following the course of the suspensory ligament, a long-neglected empyæma burrowing beneath the ventral insertion of the diaphragm, and an appendical abscess have been found as causes of this secondary involvement. The treatment must be directed towards the cure of the primary condition.

4. *Tumours of the Sub-umbilical Space that have undergone Purulent Degeneration.*—Amongst these we must mention hydatid and dermoid cysts, and primary and secondary carcinomatous deposits.

Writing on the subject of encapsulation in abdominal drainage, Frank[5] insists that drainage after cœliotomy is dangerous unless the diseased portion of the abdominal cavity can be completely shut off from the healthy. It is easy thus to isolate the pelvic cavity by utilizing the existing natural barriers, such as the meso-rectum and meso-colon, which can be made to cover the raw surface and can be further strengthened by the union of the glandulæ epiploicæ with the parietal peritoneum, so that the coils of small intestine are prevented from coming in contact with the raw surfaces. Dr. Guiseppe Amalfi[6] has brought forward an apparatus for double abdominal aspiration which he has found of use in the treatment of various forms of colic. It consists of a Y-tube, each branch of which is provided with a stop-cock, the main trunk being attached to an aspirator similar to that of Dieulafoy, and one arm is connected with the open end of an œsophageal sound, and the other with one of the extremities of an elastic tube which is attached at the other end to an ordinary rectal cannula. The apparatus is employed as follows: The œsophageal sound is passed into the stomach and the rectal cannula into the lower bowel; both stopcocks are then opened and the aspirator is put in action. If it be desired to act solely upon the upper or the lower end of the digestive tract, one of the stop-cocks is closed. By a reversed action of the aspirator an injection of gas into either the stomach or the rectum may be effected. J. D. Malcolm states[7] that the effect of carbolic acid in abdominal surgery is not so irritating to the kidneys as is popularly supposed. During the past ten years he has not seen any case in which death could be attributed to the drug. It would appear that any injury done to renal tissue by the use of carbolic acid is temporary in action and quickly recovered from, even if the secreting tissue be unhealthy, and he supports his arguments by a successful case of abdominal section for fibroma uteri in a patient suffering from advanced renal disease. R. Jones emphasises[8] the importance, in penetrating wounds of the abdomen, of following up the wound, and making it aseptic as far as possible, even though the viscera be not wounded. He refers to the statements of Dr. Dalton, who says[9] that the surgeon must explore thoroughly, and not trust the "tactus eruditus," or the patient's general condition, as an estimate of the gravity of the case, for in many fatal cases of gunshot and stab wounds there has been little appearance of shock at the time of the accident. Rydygier reports[10] a case in which a woman was relieved at one operation of two large pelvic cysts and of an enlarged and carcinomatous right ovary. In extirpating the latter the existence of adhesions made it necessary to remove a small portion of the bladder and about three inches of the sigmoid flexure. The patient recovered from the immediate effects of the operation, but, owing to symptoms of obstruction, an artificial anus was established in the ascending colon. Five weeks later, when the sigmoid flexure was found to be permeable, the artificial anus was successfully closed. The patient finally made a complete recovery.

[1] Centralbl. f. Gynak., No. 12, 1894, and Brit. Med. Journ., April 28, 1894. [2] Brit. Med. Journ., June 9, 1894, p. 1,241. [3] Berlin Klin. Woch., Nos. 4 and 5, 1894, and Med. Chronicle, June, 1894, p. 200. [4] H. Fischer (Berlin), Die Eiterungen in Subumbilicalen Raume, Sammlung Klin., Vorträge, No. 89. and Annals of Surgery, July, 1894, p. 108. [5] Central f. Gynak, 1894, No. 16, and Amer. Journ. Med. Sci., July, 1894, p. 112. [6] Med. Record, April 14, 1894, p. 469. [7] Lancet, May 5, 1894, p. 1 127. [8] Lancet, May 5, 1894, p. 1,132. [9] Journal of the Am. Med. Ass., Nov. 15, 1893. [10] Wien. Klin. Woch., No. 10, 1894, and Brit. Med. Journ., April 28, 1894.

